# Supplementary material for: Public Perception on Healthcare Services: Evidence from Social Media Platforms in China
Source: Int J Environ Res Public Health. 2019 Apr 10;16(7):1273. doi: 10.3390/ijerph16071273 (PMC6479867; doi:10.3390/ijerph16071273)
Supplement: Supplementary file 1 [file ijerph-16-01273-s001.pdf]

**Table S1: List of keywords, terms and text strings used for data searching.**

This study focuses on a list of 9 healthcare service topics. Each of these topics is represented by a service lexicon composed of service keywords and terms. There are 9 lexica, comprising 53 service keywords and terms. The first column in this table holds lexica names, subsequent columns hold service keywords and terms.

A．Chinese vision

| 主题 | 关键词 | 检索策略 |
|---|---|---|
| 就诊环境 | 环境<br>条件<br>布局<br>门诊<br>诊室<br>缴费窗口<br>诊断<br>治疗 | 医院 AND（环境 OR 条件 OR 布局）<br>医院 AND 门诊 AND（环境 OR 条件 OR 布局）<br>医院 AND 诊室 AND（环境 OR 条件 OR 布局）<br>医院 AND 缴费窗口 AND（环境 OR 条件 OR 布局）<br>医院 AND（诊断 OR 治疗）AND（环境 OR 条件 OR 布局） |
| 预约诊疗 | 预约<br>挂号<br>转诊<br>分时预约 | 医院 AND 门诊 AND（预约 OR 挂号 OR 转诊 OR 分时预约） |
| 服务效率 | 等待时间<br>就诊时间<br>治疗时间<br>检查时间<br>取药时间<br>缴费时间 | 医院 AND 门诊 AND（等待 OR 就诊 OR 治疗 OR 检查 OR 取药 OR 缴费）AND 时间 |
| 信息技术 | 微博<br>微信<br>公众号<br>服务号<br>网站<br>自助机 | 医院 AND（微博 OR 微信 OR 公众号 OR 服务号 OR 网站 OR 自助机）AND（信息 OR 服务） |
| 住院服务 | 住院<br>出院<br>入院<br>探视<br>陪护 | 医院 AND（住院 OR 出院 OR 入院）AND 服务<br>医院 AND（探视 OR 陪护）AND 条件<br>医院 AND 住院 AND（膳食 OR 饭 OR 餐） |

| | | |
|---|---|---|
| | 膳食<br>饭<br>餐 | |
| 护理服务 | 护理质量<br>护理技术<br>护士态度 | 医院 AND（护士 OR 护理）AND（质量 OR 技术 OR 态度） |
| 患者安全 | 处方<br>检查<br>检验<br>手术<br>用药<br>安全 | 医院 AND 医生 AND 处方<br>医院 AND（检查 OR 检验 OR 手术 OR 用药）AND 安全<br>医院 AND 医疗 AND 安全 |
| 人文关怀 | 医德<br>隐私保护<br>患者隐私<br>人文关怀<br>社工<br>志愿者 | 医院 AND（医生 OR 护士）AND 医德<br>医院 AND（隐私保护 OR 患者隐私）<br>医院 AND（人文关怀 OR 社工 OR 志愿者） |
| 医患关系 | 医疗纠纷<br>患者维权<br>医疗调解<br>起诉<br>诉讼<br>投诉 | 医院 AND（医疗纠纷 OR 患者维权 OR 医疗调解 OR 起诉 OR 诉讼 OR 投诉） |

B．English vision

| Topics | keywords and terms | Text strings used for searching with Boolean operators |
|---|---|---|
| Service environment | circumstances<br>environment<br>surroundings<br>outpatient service<br>consulting room<br>cashier desk<br>diagnosis<br>treatment | hospital AND (environment OR circumstances OR surroundings)<br>hospital AND outpatient service AND (environment OR circumstances OR surroundings)<br>hospital AND consulting room AND (environment OR circumstances OR surroundings)<br>hospital AND cashier desk AND (environment OR circumstances OR surroundings)<br>hospital AND (diagnosis OR treatment) AND (environment OR circumstances OR surroundings) |
| Appointment -driven service | appointment<br>registration service<br>referral<br>timeshare appointment | hospital AND outpatient service AND (appointment OR registration service OR referral OR timeshare appointment) |
| Service efficiency | waiting time<br>consultation length<br>treatment time<br>examination time<br>take the medicine time<br>payment time | hospital AND outpatient AND (waiting OR consultation OR treatment OR examination OR take the medicine OR payment) AND time |
| Information technology | Weibo<br>WeChat<br>service accounts<br>official accounts<br>website<br>Self-service machine | hospital AND (Weibo OR official accounts OR service accounts OR WeChat OR website OR Self-service machine OR App) AND (information OR service) |
| Inpatient service | inpatient<br>discharge<br>admission<br>patient visit<br>Escort | hospital AND (inpatient OR discharge OR admission) AND service<br>hospital AND patient visit AND condition<br>hospital AND inpatient AND (escort OR dietary OR food OR meal) |

| | | |
|---|---|---|
| | dietary<br>Food<br>Meal | |
| Nursing service | nursing quality<br>nursing skill<br>nurse attitude | hospital AND (nurse OR nursing) AND (quality OR skill OR attitude) |
| Patient safety | prescription<br>examination<br>test<br>operation<br>medication<br>safety | hospital AND doctor AND prescription<br>hospital AND (examination OR test OR operation OR medication) AND safety<br>hospital AND safety |
| Humanistic care | medical ethic<br>privacy protection<br>patient privacy<br>humanistic care<br>social worker<br>volunteer | hospital AND (doctor OR nurse) AND medical ethic<br>hospital AND (privacy protection OR patient privacy)<br>hospital AND (humanistic care OR social worker OR volunteer) |
| Doctor-patient relationship | medical dispute<br>patient right<br>medical mediation<br>lawsuit<br>litigation<br>complain | hospital AND (medical dispute OR patient right OR medical mediation OR lawsuit OR litigation OR complain) |
